# Targeting AKT in ER-Positive HER2-Negative Metastatic Breast Cancer: From Molecular Promises to Real Life Pitfalls?

**DOI:** 10.3390/ijms222413512

**Published:** 2021-12-16

**Authors:** Benoîte Mery, Coralie Poulard, Muriel Le Romancer, Olivier Trédan

**Affiliations:** 1 Medical Oncology Department, Centre Léon Bérard, F-69000 Lyon, France; olivier.tredan@lyon.unicancer.fr; 2 Inserm U1052, Centre de Recherche en Cancérologie de Lyon, F-69000 Lyon, France; coralie.poulard@lyon.unicancer.fr (C.P.); muriel.leromancer-cherifi@lyon.unicancer.fr (M.L.R.); 3 CNRS UMR5286, Centre de Recherche en Cancérologie de Lyon, F-69000 Lyon, France; 4 Université de Lyon, F-69000 Lyon, France

**Keywords:** estrogen-receptor-positive metastatic breast cancer, endocrine resistance, serine/threonine kinase (AKT) signaling pathway

## Abstract

The AKT protein kinase plays a central role in several interconnected molecular pathways involved in growth, apoptosis, angiogenesis, and cell metabolism. It thereby represents a therapeutic target, especially in hormone receptor-positive (HR) breast cancers, where the PI3K/AKT signaling pathway is largely hyperactivated. Moreover, resistance to therapeutic classes, including endocrine therapy, is associated with the constitutive activation of the PI3K/AKT pathway. Improved knowledge on the molecular mechanisms underlying resistance to endocrine therapy has led to the diversification of the therapeutic arsenal, notably with the development of PI3K and mTOR inhibitors, which are currently approved for the treatment of advanced HR-positive breast cancer patients. AKT itself constitutes a novel pharmacological target for which AKT inhibitors have been developed and tested in clinical trials. However, despite its pivotal role in cell survival and anti-apoptotic mechanisms, as well as in endocrine therapy resistance, few drugs have been developed and are available for clinical practice. The scope of the present review is to focus on the pivotal role of AKT in metastatic breast cancer through the analysis of its molecular features and to discuss clinical implications and remaining challenges in the treatment of HR-positive metastatic breast cancer.

## 1. Introduction

Breast cancer is the leading cause of cancer-related deaths in women worldwide. Though longer life spans and decreased rates of breast cancer-specific mortality have been achieved for localized stage tumors thanks to advances in population screening and early-stage treatments, metastatic breast cancer remains an incurable disease. Indeed, despite major advances in the treatment of metastatic breast cancer and improved therapies, most patients die of their disease [1]. Breast cancer is a heterogeneous disease for which a molecular classification has been compiled based on the expression of biomarkers, including estrogen/progesterone receptors (ERα/PR) and human epidermal growth factor receptor-2 (HER2) [2,3]. ERα/PR-positive luminal A and B tumors are the most predominant in comparison to HER2-enriched and triple-negative breast cancers (TNBCs). Luminal A tumors are typically low-grade and associated with better prognosis in comparison to luminal B tumors [4]. According to this classification, different treatment strategies are required. For these luminal tumors in particular, endocrine therapy, in combination with cyclin-dependent kinase 4 and 6 inhibitors (CDK4/6), is now the cornerstone of first-line therapy in the metastatic setting, especially when the estrogen receptor is highly expressed [5]. Endocrine therapies administered to pre-menopausal patients include molecules promoting suppression of ovarian function (gonadotropin-releasing hormone agonists, such as goserelin) combined with non-steroidal aromatase inhibitors (anastrozole or letrozole), or steroidal aromatase inhibitors such as exemestane. The selective ER modulator (tamoxifen) and selective ER down-regulator (fulvestrant) are also routinely used [6].

Aside from molecules used in endocrine therapy, several targeted agents have emerged [7]. Indeed, a better understanding of resistance mechanisms to endocrine therapy has led to the development of new drugs targeting pathways involved in cell proliferation, albeit estrogen deprivation. Resistance to endocrine therapy is inevitably encountered, likely with the activation of alternative pathways allowing cancer cells to survive [8]. In particular, dysregulation of the phosphoinositide 3 kinase (PI3K)/AKT/mammalian target of the rapamycin (mTOR) pathway is one of the major intracellular signaling pathways of secondary endocrine resistance, thus prompting the development of PI3K pathway inhibitors [9]. The majority of breast cancers display an alteration in the PI3K/AKT pathway, most frequently through somatic hotspot mutations in exons 9 and 20 of PIK3CA, leading to cell growth, proliferation, angiogenesis, and cell survival. Such mutations encode the p110α isoform of PI3K [10]. The development of mTOR and PI3K inhibitors, such as everolimus and alpelisib, has led to a substantial increase in progression-free survival (PFS). Indeed, the BOLERO-2 trial evaluating everolimus, a selective mTOR inhibitor, in addition to exemestane, reported an improvement in PFS, but not in overall survival [11]. The SOLAR-1 trial evaluated a PI3Kα-specific inhibitor, alpelisib, and reported a prolonged PFS among patients harboring PIK3CA-mutated, HR-positive, HER2-negative advanced breast cancers who had previously received endocrine therapy [12]. Another phase III trial involving buparlisib, a pan-PI3K inhibitor targeting all four isoforms of class I PI3K (α, β, γ, δ) in combination with endocrine therapy (fulvestrant), was also published, presenting data on the efficacy of the inhibitor on patients with PIK3CA mutations. However, this study was not further extended owing to the lack of favorable safety profile [13]. Beyond the development of PI3K inhibitors, targeting ATK through its inhibition is also currently explored [14,15]. Indeed, AKT mutations are involved in the hyperactivation of the PI3K pathway, and AKT plays a crucial role in interconnected cell signaling mechanisms. However, if in vitro and in vivo models of AKT inhibition have been developed, few drugs have entered clinical evaluation, and none has been yet approved in the therapeutic arsenal for metastatic breast cancer [16]. Understanding the gap between in vitro studies and clinical practice with regard to AKT inhibition is of paramount importance as no AKT inhibitor is available for the management of patients, despite its pivotal role in molecular signaling pathways and endocrine resistance. The scope of the present review is to focus on the pivotal role of AKT in estrogen-receptor-positive, HER2-negative metastatic breast cancer from a molecular point of view; we will subsequently present the molecular features of the AKT pathway and its involvement in endocrine therapy resistance before discussing current and perspective treatments in clinical practice through the promises and pitfalls encountered in clinical practice.

## 2. Akt Kinase Pathway: Molecular Features

**Figure 1:**
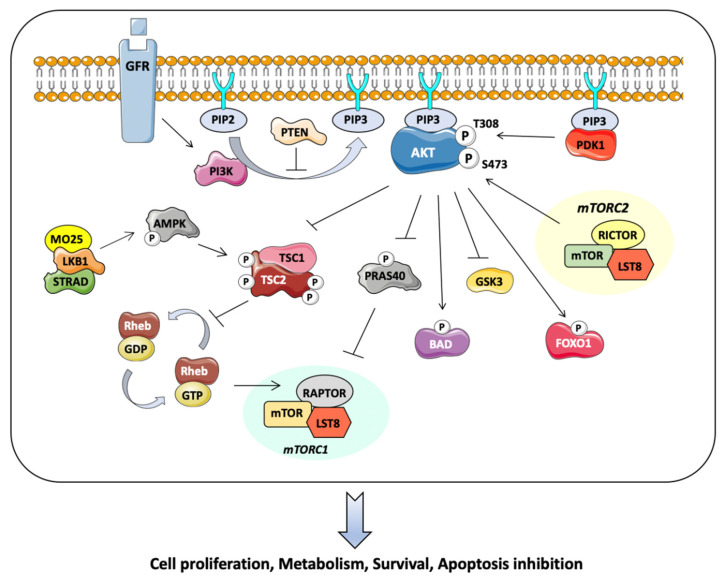
Central role of AKT in the Pi3K/AKT/mTOR pathway in breast cancer.

The serine/threonine kinase AKT also known as protein kinase B has a central role in interconnected cell signaling mechanisms involved in cell growth, apoptosis, and angiogenesis. Three isoforms of AKT (AKT1, AKT2, and AKT3) are described, with similar conserved protein structures. AKT displays three domains, including an amino-terminal fragment (N-terminal), a central fragment, and a carboxyl-terminal fragment (C-terminal). The kinase domain displays a regulatory threonine residue Thr308, the phosphorylation of which leads to the activation of AKT. A regulatory serine residue, Ser473, is present in the C-terminal fragment and constitutes the other main phosphorylation site of AKT [17] (Figure 1). With regard to the distribution of the three isoforms, AKT1 and AKT2 are ubiquitous, whereas AKT3 is mostly expressed in neural cells. Activation of these isoforms (AKT1, AKT2, and AKT3) has been linked to tumor progression through the regulation of cellular growth factors and the blockade of pro-apoptotic proteins [18]. AKT is central to the PI3K/AKT/phosphatase and tensin homolog (PTEN)/mTOR pathway, which is activated in almost 70% of breast cancers through mutations involving Akt1, PTEN, and PI3K. The PI3K catalytic alpha subunit mutation (PIK3CA), for instance, has been detected in approximately 40% of patients with hormone-receptor-positive breast cancer [19]. Loss of function of PTEN has been described in 35% of cases, and AKT substitutions and amplifications are present in 5% to 10% of cases. As such, the most important negative regulator of AKT remains PTEN, as it constitutes an antagonist of PI3K, leading through its phosphatase activity to the dephosphorylation of phosphatidyl-inositol-3,4,5-triphosphate (PIP3) to phosphatidyl-inositol-4,5-biphosphate (PIP2) (Figure 1). Subsequently, the loss of function of PTEN leads to enhanced PI3K signaling with decreased apoptosis and loss of cell growth control [20]. Other alterations that can activate AKT signaling are phosphoinositide-dependent kinase (PDK1) amplification, under-expression of inositol polyphosphate 4-phosphatase II (INPP4B), and under-expression of liver kinase B (LKB1) [21] (Figure 1). AKT1 was recently shown to be regulated by methylation through its symmetric dimethylation by the arginine methyltransferase PRMT5 (protein arginine methyltransferase 5) on arginine 391. This activation of AKT, in turn, leads to its translocation at the level of the plasma membrane [22].

The Pi3K/AKT/PTEN/mTOR pathway is activated by several growth factor receptors, including HER2, fibroblast growth factor receptor 1 (FGFR1), and insulin-like growth factor 1 (IGF1R). In addition, estrogen has also been shown to activate PI3K/AKT signaling by a direct binding of cytoplasmic ERα to PI3K and Src. This occurs following ERα methylation on the arginine 260 residue by PRMT1, another member of the arginine methyltransferase family performing asymmetric demethylation [23]. Interestingly, ERα/Src and ERα/PI3K interactions are correlated with the activation of the downstream effector AKT in a subset of breast tumors. Survival analysis revealed that high expression of the ERα/PI3K/Src complex is an independent marker of poor prognosis and associated with reduced disease-free survival [24,25]. This methylation event also plays a crucial role in IGF1 signaling, as PRMT1 impairs IGF1R binding to ERα and downstream AKT signaling [26]. Mechanistically, activated PI3K catalyzes the conversion of PIP2 into the second messenger PIP3. The recruitment to the plasma membrane of AKT and PDK1 by PIP3 leads to the double phosphorylation of AKT on the kinase domain and the regulatory domain by the mTOR complex. The activation of AKT ultimately leads to the phosphorylation of a large number of targets, such as tuberos sclerosis complex 2 (TSC2), glycogen synthase kinase-3β (GSK3β), and the transcription factors of the forkhead box, class O (FOXO) involved in cell proliferation, metabolism, and survival [27] (Figure 1). Activation of the TSC2 complex leads to the inhibition of pro-apoptotic proteins and signals with the dissociation of the TSC1-TSC2 complex; such a dissociation ultimately activates TORC1, resulting in the inhibition of autophagy [11]. With regard to pro-apoptotic signals, including FOXO transcription factors, they are inactivated by AKT through their nuclear exclusion, resulting in cell cycle progression. In parallel, the extrinsic apoptotic pathway involving Fas Ligand (FasL) and TNF-related apoptosis-inducing ligand (TRAIL) is also inhibited [28,29]. Actors of the PI3K/AKT/PTEN/mTOR pathway with the central role of AKT are depicted in Figure 1.

## 3. Inhibition of AKT in Early Clinical Trials

Dysregulations of the PI3K/AKT pathway in hormone-receptor-positive breast cancer constitute oncogenic drivers and are implicated in resistance to treatment. Recent molecular profiling data indicated that a PIK3CA mutation in this subtype of metastatic breast cancer leads to resistance to chemotherapy and endocrine therapy, and to poor patient outcome [30]. Currently, molecules targeting mTOR and PI3K represent the main therapeutic options for metastatic breast cancer patients after first-line CDK4/6 inhibitor-based therapy, given the results obtained through the BOLERO II and SOLAR I trials [12]. In the SOLAR-I trial, all patients were considered endocrine-resistant, as they relapsed during or within the 12 months following its completion. Patients of the BOLERO II trial were also considered an endocrine-resistant trial as they had relapsed or progressed during endocrine therapy [11,12]. However, it has been suggested that if the inhibition of PI3K/AKT/mTOR results in a reduction of cell proliferation and survival, the concomitant activation of compensatory mechanisms occurs, conferring cells with resistance to single inhibitors that may explain progression of disease in the BOLERO-II and SOLAR-I trials [31]. For instance, mTOR inhibition results in activation of AKT and extracellular signal-regulated kinase (ERK), leading to increased signaling through those branches of the pathway [32]. Upstream tyrosine kinases are activated by the inhibition of PI3K, leading to cell proliferation without the inhibitory effects expected by the blockade of PI3K. The activation of AKT by PI3K allows the phosphorylation of ERα at S167, resulting in an independent activation of ERα-mediated transcription [33]. In vitro studies demonstrated that the ERα pathway can be activated by AKT independently of estrogen availability [34]. Subsequently, one of the major mechanisms of endocrine resistance has been elucidated, notably the PI3K/AKT/mTOR pathway activation through cross-talk with estrogen-mediated signaling [35].

Moreover, AKT alterations are present in approximately 7% of hormone-receptor positive breast cancers, the predominant mutation being AKT1^E17K^ since it represents 80% of cases. The importance of AKT in breast cancer and its involvement in resistance to endocrine therapy have made it a much-pursued target for anticancer therapy with the aim of developing selective and potent AKT inhibitors. Within this context, several AKT inhibitors have been synthetized and tested in early clinical trials in HR-positive metastatic breast cancer, showing preliminary signs of efficacy. One of them, capivasertib (AZD5363) is an oral ATP-competitive pan-AKT kinase inhibitor associated with an anti-proliferative activity in preclinical models [36]. Ribas et al. demonstrated that inhibition of AKT with capivasertib re-sensitizes cells to tamoxifen, in combination with fulvestrant, preventing the emergence of hormone-independent cells in vivo. It is interesting to note that, in their study, the inhibition of AKT was associated with positive feedback loops driven by MYC, leading to increased gene expression of human epidermal receptor growth factor 2 and 3 (ERBB2-ERBB3), ERK5, and IGF1 [37]. Such results may suggest the need for combining multiple inhibitors. In a phase I trial, the combination of capivasertib and fulvestrant among heavily pre-treated patients with HR-positive and an activating AKT1^E17K^ mutation resulted in an objective response rate (ORR) of 30% in fulvestrant pre-treated patients and 20% in fulvestrant-naïve patients, whereas the ORR with monotherapy was 20% [17]. Such low results are consistent with the fact that efficacy in HR-positive breast cancer may be limited by a compensatory increase in HR-dependent gene transcription, hence the need for combination strategies in order to maximize therapeutic efficacy [38,39]. With regard to tolerance, it appears that the combination therapy was more tolerable than monotherapy, with fewer rashes, hyperglycemia, and diarrhea, as the dose of capivasertib was lower in the combination therapy group. These data argue in favor of considering AKT as a clinically relevant target and capivasertib as an active, well-tolerated drug among patients displaying endocrine resistance. Of note, the search for detectable AKT1^E17K^ mutations was feasible in clinical practice through centralized plasma screening. The highly potent and allosteric pan-AKT inhibitor MK-2206 has also been associated with similar results in another phase I trial, in combination with fulvestrant, anastrozole, or both, among patients with HR-positive, HER2-negative breast cancer [40]. MK-2206 was administered orally on a weekly basis at a dose of 150 mg, in combination with prednisone used as a prophylaxis. Clinical benefits without progression within 6 months were observed for 42% of patients. This trial was developed on the basis of in vitro studies demonstrating that MK-2206 induced apoptosis in ER-positive breast cancer cells under estrogen-deprived conditions or in combination with fulvestrant [41]. However, high doses of MK-2206 were not administered due to skin toxicity with grade 3 rashes for 20% of patients and grade 3 hyperglycemia in 30% of cases. The administration of MK-2206 has been investigated in several clinical trials beyond breast cancer with proven efficacy in vitro on cancer cells, with apoptotic effects.

However, it seems that the use of MK-2206 in clinical practice through clinical trials is limited by its skin toxicity. The administration of MK-2206 at 60mg has led to several toxicities, including grade 3 maculopapular rash in 1 in 2 patients; consequently, efficacy may be limited by toxicity, with the need to reduce the administration dose [42,43]. In the phase Ib/II BEECH trial, patients received capivasertib or a placebo in combination with weekly paclitaxel in situations of endocrine resistance. PFS was 10.9 months with capivasertib, versus 8.4 months in the placebo group (HR = 0.80, 80% CI (0.60–1.06) *p* = 0.308). In patients with PIK3CA mutations, the results were not improved despite strong preclinical data in this sub-population of patients [44]. So far, it seems that the translation of in vitro studies into clinical practice concerning the benefits of AKT-targeted therapies may be insufficient in the Erα-positive breast cancer setting. Further investigations are required through phase III trials to extend data on AKT inhibitors [16]. Preliminary promising results of a phase Ib trial were reported in a 2020 ASCO meeting evaluating the combination of ipatasertib and endocrine therapy with or without palbociclib in patients with HR-positive metastatic breast cancer progressing after first-line CDK4/6 treatment. Partial responses and stable diseases were observed in the group with ipatasertib as AKT alterations may confer resistance to CDK4/6 inhibition [45]. Cell cycle progression is regulated by cyclins, as well as cyclin-dependent kinases, and cyclin D1, through its binding to CDK4/6, allows the regulation of the G1 phase of the cell cycle. Cyclin D1 is a downstream target of the PI3K/AKT/mTOR pathway [46]. Cross-talk between CDK4/6 and the ER signaling pathways has also been demonstrated. Indeed, inefficacy of PI3K inhibitors, likely due to persistent phosphorylation of the retinoblastoma (RB) protein, could be counterbalanced by their combination with a CDK inhibitor to overcome PI3K inhibitors insensitivity [47]. If the use of CDK4/6 inhibitors is associated with significant clinical benefits in combination with endocrine therapies, and is currently used in the first-line setting of ER-positive, HER2-negative metastatic breast cancer, such drugs mostly induce tumor stabilization with limited cytostatic effects because of primary and secondary resistance [48]. It has been demonstrated that such resistance was mediated by loss of the RB1 gene [49]. In this context, the combination of CDK4/6 and AKT inhibitors may be relevant as evidenced in the phase Ib trial reported in the 2020 ASCO meeting, but require further and more robust studies. Results of clinical trials involving AKT inhibitors in HR-positive breast cancer are presented in Table 1.

## 4. Clinical Practice and Therapeutic Perspectives

Among more advanced trials involving AKT inhibitors, the FAKTION phase II study evaluated whether the addition of capivasertib to fulvestrant improved PFS in patients with advanced breast cancer in the context of endocrine resistance. It was a randomized, double-blind, placebo-controlled study encompassing 183 patients who were assigned to receive fulvestrant plus capivasertib or fulvestrant plus placebo. Pre-menopausal women and men were excluded. No patients had received previous CDK4/6 inhibitors. Median PFS was significantly longer for patients who received capivasertib (10.3 months in the capivasertib group versus 4.8 months in the placebo group (HR = 0.58 (95% CI (0.39–0.84)) *p* = 0.0017), with a statistically significant gain in 5.5 months. However, this benefit was not seen in PI3K/PTEN-altered tumors. This was the first randomized trial to report an improvement in PFS and response rate of an AKT inhibitor combined to endocrine therapy in ER-positive, HER2-negative metastatic breast cancer, regardless of the PI3K pathway alteration status. Of note, adverse events, such as hypertension, rash, diarrhea, and hyperglycemia, were common but manageable with dose reduction and did not compromise efficacy. More than a third of patients (41%) had at least one dose reduction and only three patients stopped capivasertib because of toxicity. A randomized phase III trial (CAPItello-291) investigating the association of capivasertib and fulvestrant is currently ongoing in HR-positive, HER2 metastatic breast cancer patients in order to confirm the data of the FAKTION study, in the context of endocrine resistance [50]. It is worth underlining that, in the FAKTION study, no patients had received previous CDK4/6 inhibitors, which are now the standard-of-care in combination with endocrine therapy in the first-line setting of HR-positive, HER2 metastatic breast cancer. However, preclinical data suggest that the benefit of using capivasertib should not be lost in the event of previous exposure to CDK4/6 inhibitors [51]. Moreover, preclinical data suggest the superiority of AKT inhibition in comparison to PI3K inhibition, which needs to be explored and confirmed in a randomized trial [52].

Ipatasertib, another AKT inhibitor which was initially tested in TNBC, has been evaluated in HR-positive, HER2-negative advanced breast cancers through the IPATunity 130 randomized phase III trial, among patients with PIK3CA/AKT1/PTEN altered tumors. Patients were assigned to receive ipatasertib plus paclitaxel, or paclitaxel plus placebo. Median PFS was 9.3 months for both arms, without improvement in efficacy with the therapeutic combination [53]. Another phase III trial focusing on the combination of ipatasertib and fulvestrant in metastatic HR-positive, HER2-negative breast cancer, in the context of progression after a first-line therapy with aromatase inhibitor and cyclin-dependent kinase 4/6 inhibitor, is ongoing to reinforce data on this AKT inhibitor [54]. Another phase III randomized trial called IPATunity150 is currently evaluating the efficacy of the combination of ipatasertib, fulvestrant, and palbociclib in comparison with placebo, fulvestrant, and palbociclib as first-line treatment among patients with endocrine-resistant breast cancers naïve to CDK4/6 inhibitors [55]. Only a few data are available on early stages of HR-positive breast cancer. MK-2206 was administered in a neo-adjuvant setting in combination with anastrozole through a phase II trial, which was closed prematurely owing to the lack of pathological complete response [56]. This drug was also tested with standard pre-operative therapy in the phase II I-SPY2 trial, but pathological complete response was not significantly improved [57].

With regard to tolerance, inhibition of the PI3K/AKT pathway is associated with a specific profile of toxicity often manageable in clinical practice. However, the safety of AKT inhibitors may be a limiting factor for their development [58]. In particular, skin toxicity is a major concern with AKT inhibitors likely due to the involvement of PI3K/AKT in keratinocyte differentiation [59]. All AKT inhibitors were associated with such toxicity, notably with a grade 3 toxicity almost reaching 30% with MK-2206. The management of such toxicity often requires topical or systemic steroids with drug interruption, dose reduction, and, in some cases, treatment discontinuation. Premedication with prednisone was not highly effective with regard to the management of skin toxicity. Digestive toxicity with diarrhea was the most common adverse event reported with AKT inhibitors, requiring dose reductions and treatment discontinuations. Most of the cases were moderate, with few grade 3 events. Symptoms were reversible using antidiarrheal agents. Hyperglycemia ranged from 4% with ipatasertib to 92% with MK-2206, as insulin-mediated glucose homeostasis mainly relies on PI3K signaling. Capivasertib and ipatasertib were associated with a high incidence of grade 3 hyperglycemia without further complications, such as hyperosmolar coma. Treatment involved treatment interruption with dose reduction, in combination with dietary intervention and medication for diabetes. Hepatic toxicity, hypertension, stomatitis, and dyslipidemia occurred less frequently in comparison with other inhibitors of the PI3K/AKT pathway [60]. Consequently, if AKT inhibitors constitute potential new drugs in the fight against ER-positive metastatic breast cancer, further studies are warranted with regard to the management of adverse events in clinical practice. Moreover, it is of paramount importance to identify predictive biomarkers of the response to AKT inhibitors in order to improve the efficacy of these targeted agents in a “personalized medicine” therapeutic approach. Translational studies have been conducted, but results are not conclusive. On the one hand, preclinical data suggest that alterations in the PI3K/AKT pathway shown previously may sensitize breast cancer cells to AKT inhibitors, whereas, on the other hand, a lack of correlation between PI3K/AKT pathway alterations and efficacy of AKT inhibitors seems to emerge from recent trials conducted in HR-positive breast cancer patients [36,40]. Subsequently, several strategies need to be elaborated in order to improve the efficacy of AKT inhibitors beyond predictive biomarkers. Impairment of AKT alone is likely not sufficient, hence the need to combine AKT inhibitors with other targeted therapies, such as PRMT inhibitors, in order to block cancer cell survival and proliferation, as well as other compensatory pathways. Combining AKT and mTOR inhibitors may also be relevant to overcome drawbacks encountered with the use of AKT inhibitors without combination. A better selection of patients with *AKT* mutations may also be useful to improve efficacy. Other PI3K pathway alterations in HR-positive advanced breast cancer also need to be explored. Ongoing trials on AKT inhibitors in HR-positive breast cancers are presented in Table 2.

## 5. Conclusions

This review underlines the implication of AKT in ER-positive, HER2-negative metastatic breast cancer biology. AKT plays a crucial role in resistance to endocrine therapy in HR-positive metastatic breast cancer, prompting the development of several therapeutic strategies based on AKT inhibitors. Preclinical data using cell lines and xenograft models of AKT inhibition led to the launching of phase III clinical trials. However, several challenges remain as no drug has so far entered clinical practice. Indeed, our review underlines discrepancies between in vitro results and clinical trials and the difficulty in developing AKT inhibitors in clinical practice despite its pivotal role in molecular pathways. Consequently, future research perspectives will require the evaluation of new combinations in order to overcome resistance mechanisms, as well as the development of predictive biomarkers for a better selection of patients. In the context of endocrine resistance, cross-talk between the PI3K/AKT/mTOR and ER pathways has major clinical relevance, but further investigations are required to completely understand the interaction between these pathways and develop drugs to block cell proliferation. In addition, reducing adverse effects remains a goal for AKT inhibitors, as it could represent the major limitation to their use. Indeed, AKT inhibitors might be limited by adverse events related to a spectrum of off-target effects. Beyond data efficacy, the essential condition for prescribing AKT inhibitors in clinical practice remains the safety profile. The need for combining AKT inhibitors with other molecules to ensure their efficacy may also increase toxicity and, subsequently, may not be suitable for clinical applications. In conclusion, despite major breakthroughs in our understanding of ER-positive, HER2-negative metastatic breast cancer, and especially mechanisms of resistance to treatment, several challenges in this field of research still remain.

## Figures and Tables

**Table 1:** Results of clinical trials involving AKT inhibitors in HR-positive breast cancer.

AKT Inhibitor	Trials	Study Treatment	Phase and Study Design	Setting	Results
CAPIVASERIB	NCT01226316	Capivasertib +/− fulvestrant among patients with AKT1^E17K^ mutation	I–open label	Metastatic breast cancer	ORR = 30% in the combination group versus 20% with monotherapy
	BEECH NCT01625286	Capivasertib or placebo + paclitaxel	Ib/II-randomized, double-blind	Metastatic breast cancer	PFS = 10.9 months with capivasertib versus 8.4 months with placebo (HR = 0.8, 80% CI (0.6–1.06) *p* = 0.308)
	FAKTION NCT01992952	Capivasertib or placebo + fulvestrant	Ib/II, randomized, double-blind	Metastatic breast cancer	PFS = 10.3 months with capivasertib versus 4.8 months with placebo (HR = 0.58 (95% CI (0.39–0.84)) *p* = 0.0017)
IPATASERTIB	IPATunity 130 NCT03337724	Ipatasertib + paclitaxel or paclitaxel + placebo	III, randomized	Metastatic breast cancer	PFS = 9.3 months in both arms
	TAKTIC NCT03959891	Ipatasertib + fulvestrant + palbociclib or placebo	Ib	Metastatic breast cancer	Clinical benefit for 8/12 patients with partial response and stable disease
MK-2206	NCT01344031	MK-2206 + anastrozole and/or fulvestrant	I, open label dose finding	Metastatic breast cancer	Clinical benefit within progression for 42% of patients

PFS: progression-free survival; ORR: objective response rate.

**Table 2:** Ongoing clinical trials involving AKT inhibitors in HR-positive breast cancer.

AKT Inhibitor	Trials	Study Treatment	Phase and Study Design	Setting
CAPIVASERIB	CAPItello-291 NCT04305496	Capivasertib + fulvestrant versus placebo + fulvestrant	III-randomized, double-blind	Metastatic breast cancer in context of endocrine resistance
	NCT03310541	Capivasertib + fulvestrant	I-multi-cohort, non randomized	Metastatic breast cancer, with AKT mutation, in context of endocrine resistance
IPATASERTIB	IPATunity 150 NCT04060862	Ipatasertib + palbociclib + fulvestrant or placebo + palbociclib + fulvestrant	III-randomized, double-blind	Metastatic breast cancer, endocrine resistant, naive to cdk4/6 inhibitors
	NCT03280563	Ipatasertib + atezolizumab or fulvestrant	Ib-II-multi-cohort, randomized	Metastatic breast cancer, after CDK4/6 inhibitor
	NCT03395899	Ipatasertib+ atezolizumab or ipatasertib + bevacizumab + atezolizumab or atezolizumab	II-multicohort, randomized	Metastatic breast cancer

## Data Availability

Not applicable.
